# On a roll: a direct comparison of extraction methods for the recovery of eDNA from roller swabbing of surfaces

**DOI:** 10.1186/s13104-023-06669-5

**Published:** 2023-12-18

**Authors:** Austin M. Guthrie, Paul Nevill, Christine E. Cooper, Philip W. Bateman, Mieke van der Heyde

**Affiliations:** 1 MBioMe - Mine Site Biomonitoring Using eDNA Research Group, Trace and Environmental DNA (TrEnD) Laboratory, School of Molecular and Life Sciences, Curtin University, Perth, WA 6102 Australia; 2 School of Molecular and Life Sciences, Curtin University, Perth, WA 6102 Australia; 3 Behavioural Ecology Laboratory, School of Molecular and Life Sciences, Curtin University, Perth, WA 6102 Australia

**Keywords:** Environmental DNA, eDNA, DNA extraction

## Abstract

**Objective:**

Roller swabbing of surfaces is an effective way to obtain environmental DNA, but the current DNA extraction method for these samples is equipment heavy, time consuming, and increases potential contamination through multiple handling. Here, we used rollers to swab a dog kennel and compared three DNA extraction approaches (water filtration, roller trimming and direct buffer) using two different platforms (Qiacube, Kingfisher). DNA extraction methods were evaluated based on cost, effort, DNA concentration and PCR result.

**Results:**

The roller trim method emerged as the optimal method with the best PCR results, DNA concentration and cost efficiency, while the buffer-based methods were the least labour intensive but produced mediocre PCR results and DNA concentrations. Additionally, the Kingfisher magnetic bead extractions generally ranked higher in all categories over the Qiacube column-based DNA extractions. Ultimately, the ideal DNA extraction method for a particular study is influenced by logistical constraints in the field such as the size of the roller, the availability of cold storage, and time constraints on the project. Our results demonstrate the strengths and weaknesses of each approach, allowing for informed decision making by researchers.

**Supplementary Information:**

The online version contains supplementary material available at 10.1186/s13104-023-06669-5.

## Introduction

Environmental DNA (eDNA) surveys have emerged as a useful tool for monitoring ecosystem biodiversity, including rare and cryptic species that can be difficult to detect using conventional methods such as live trapping or the use of remote cameras [1]. Recently, sampling vegetation surfaces including bark [2], foliage [3], tree hollows [4] and fruit [5] for eDNA has been used to detect terrestrial fauna. Methods of capturing eDNA from these surfaces include spray aggregation, which involves collecting water that is washed over the sampling surface [2, 3]; forensic swabs, which are dipped in water and swabbed over the sampling surface [6, 7]; and roller swabs, which are dampened by water and rolled across the sampling surface [8]. The use of roller swabs holds promise as it allows for more comprehensive sampling of surfaces than forensic swabs and requires a smaller volume of water than spray aggregation, facilitating sampling at remote sites. However, current techniques for extracting eDNA from roller swabs are time consuming and rely upon the resuspension of eDNA from rollers using water and subsequent filtration prior to extraction [4]. These additional steps may introduce contamination [9] while also increasing the amount of equipment needed and the time required to process large batches of samples [10]. Therefore, there is a need for a simplified extraction method for DNA from roller swabs. Here we compare three methods (water filtration, roller trimming and direct buffer) to process domestic dog eDNA from roller swabs and two protocols to extract DNA from the digests (a column-based and a magnetic bead extraction protocol) with the aim of optimising the analysis of roller swab samples in terms of the quality of results, effort and cost parameters.

## Methods

### Roller method protocols

A kennel used by a medium-sized domestic dog (*Canis lupus familiaris*) was sampled with a roller technique adapted from Valentin et al. (2020), using decontaminated 50 mm microfibre rollers dampened with deionised water (See Additional Information for details). The kennel, approximately 100 cm (L) × 70 cm (W) × 70 cm (H), was sampled systematically on all interior surfaces, ensuring each sample did not overlap a previously sampled area, resulting in a total of six roller samples. A control sample was also taken by exposing a damp roller to the air around the kennel without rolling. Each sample was stored on ice in a zip lock bag, frozen within 12 h of sampling and stored at -20°C prior to extraction. Each roller was sliced into thirds within a sterile fume hood and individually stored in 50 mL falcon tubes maintained at − 20 °C. These samples were then randomly assigned to one of the following DNA processing methods (see below), and their resulting digests assigned one of two extraction methods. Two extraction controls were processed simultaneously for all four methods using reagents only.

### Processing methods

*Filtration method*: 40 mL of deionised water was added to each falcon tube and agitated on a Tissuelyser (Qiagen, Hilden, Germany) for ten minutes at 10 rps. The section of roller was then removed, and the resulting water samples filtered across a Pall 0.45 µm GN-6 Metricel® mixed cellulose ester membrane using a peristaltic Pall Sentino^®^ Microbiology pump (Pall Corporation, Port Washington, USA) and frozen at − 20 °C prior to extraction. Half of each filter membrane was digested in 540 µL of ATL lysis buffer and 60 µL of Proteinase K for 18 h at 56 °C.

*Roller trim method*: A section of the roller segment was trimmed into a 2 mL Eppendorf tube using sterile scissors. Each sample was digested for 18 h at 56 °C by adding 1000 µL of ATL lysis buffer and 60 µL of Proteinase K.

*Buffer method*: 15 mL of a 25% MagMax Microbiome Lysis Solution (ThermoFisher Scientific) was added to each falcon tube containing the roller section and agitated on a Tissuelyser for five minutes at 10 rps. Falcon tubes were then stored at 4 °C for approximately seven days, before 40 µL of Proteinase K was added to 560 µL aliquots of each sample and digested for either 20 min (herein buffer 20 min) or overnight (18 h, herein buffer 18 h).

### Extraction methods

DNA was extracted from digesta via two methods: a column-based DNA extraction protocol (DNeasy Blood and Tissue Kit, Qiagen) and a magnetic bead extraction protocol (MagMAX Microbiome Ultra Nucleic Acid Isolation Kit, Applied Biosystems, USA). Both methods used between 400–500 µL of DNA digest and produced 50–100 µL of DNA extract. With four processing methods (filtration, roller trim, and two from the buffer method), two extraction platforms, and 6 replicates there was a total of 48 samples (Additional file 1: Figure S1). *Analysis of extracts.*

Following extraction, total DNA concentrations for all samples were recorded using a QuBit fluorometer (Thermo Fisher Scientific). Quantitative polymerase chain reaction (qPCR; Applied Biosystems, USA) with a dog specific primer [11] was used to assess the quality and quantity of the DNA extracts (See Additional Information for details).

### Statistical analysis

All statistical analyses were performed using IBM SPSS Statistics (Version 25). Multivariate repeated measures ANOVAs were conducted to evaluate the effect of extraction method on both mean cycle threshold (Ct) values and DNA extraction concentrations, with processing method (filtration, roller trim, buffer 20 min or buffer 18 h) and extraction method (Qiacube or Kingfisher) as two within-group factors and the individual roller as the repeat. Simple *a-priori* contrasts [12] were used to determine where significant differences were present, using filtration (the standard extraction method for rollers; [2]) as the comparison group.

### Method ranking

Each combination of methods (n = 8) was ranked across four categories: Ct value, DNA concentration, cost and effort. Ct values from qPCRs were ranked from lowest (rank 1) to highest value (rank 8). Total DNA concentrations were ranked from highest (rank 1) to lowest concentration (rank 8). A total cost of analysis per sample for each method was calculated using costings of reagents, sampling equipment and other consumables available at the time of the study (Additional file 1: Figure S2) and ranked from least (rank 1) to most expensive (rank 8). The approximate effort, measured as total processing time per sample (Additional file 1: Figure S3), was also given a rank from lowest (rank 1) to highest effort (rank 8).

## Results

**Fig. 1:**
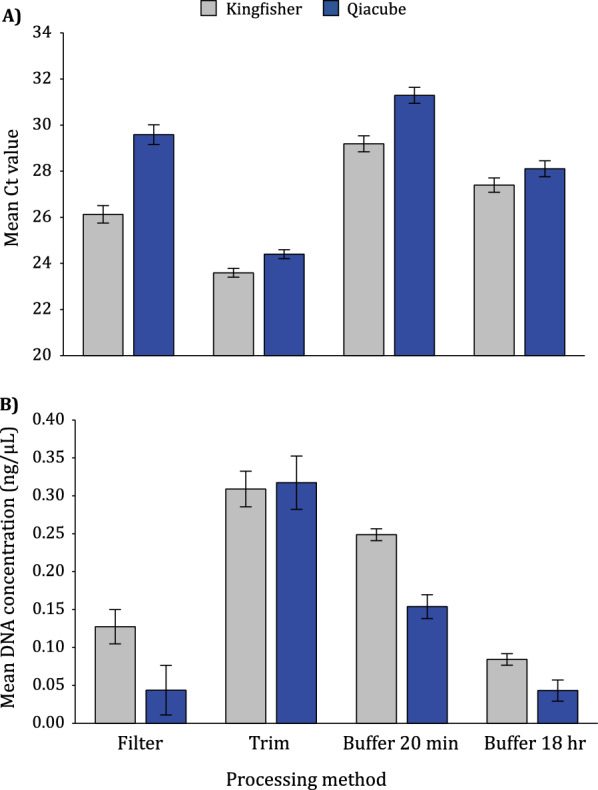
**A** Cycle threshold (C_t_) values and **B** total DNA concentration for roller swab samples taken from a domestic dog kennel and extracted using four processing methods and two extraction methods. Values are mean ± SE, n = 6 per treatment

Sample processing method (F_3,3_ = 107, P = 0.002) and extraction method (F_1,5_ = 296, P < 0.001) significantly impacted mean Ct values (Fig. 1), with a significant interaction between the effects of processing and extraction method on Ct values (F_3,3_ = 3006, P < 0.001). Post-hoc contrasts revealed that mean Ct values for trim (Q: 24.4 ± 0.20; K: 23.6 ± 0.20) and buffer 20 min (Q: 31.3 ± 0.35; K: 29.2 ± 0.34) techniques differed significantly from the conventional filtration approach (Q: 29.6 ± 0.43; K: 26.2 ± 0.38; F_1,5_ ≥ 27.9, P < 0.003), while the Ct values for the buffer 18 h approach were not significantly different (Q: 27.4 ± 0.32; K: 28.1 ± 0.35; F_1,5_ = 0.035, P = 0.859). Samples extracted via the Kingfisher had consistently lower Ct values than those extracted by the Qiacube.

Total DNA concentration also differed significantly between processing (F_3,3_ = 104, P = 0.002) and extraction methods (F_1,5_ = 27.2, P = 0.003; Fig. 1). Post-hoc contrasts revealed that DNA concentrations for trim (Q: 0.32 ± 0.035 ng µL^−1^; K: 0.31 ± 0.024 ng µL^−1^) and buffer 20 min (Q: 0.15 ± 0.016 ng µL^−1^; K: 0.25 ± 0.008 ng µL^−1^) techniques differed significantly from the conventional filtration approach (Q: 0.04 ± 0.033 ng µL^−1^; K: 0.13 ± 0.023 ng µL^−1^; F_1,5_ ≥ 22.7, P < 0.005), while the buffer 18 h did not (Q: 0.043 ± 0.014 ng/µL; K: 0.084 ± 0.008 ng/µL; F_1,5_ = 1.19, P = 0.325).

**Fig. 2:**
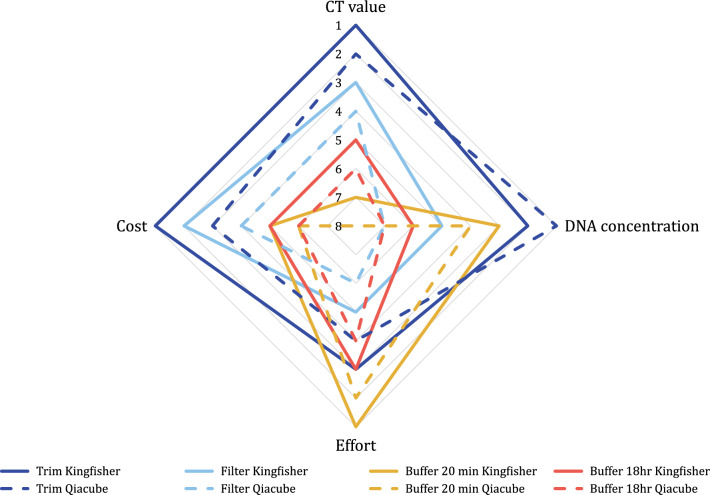
Ranked radar plot assessing combinations of processing and extraction methods for roller swabs, based on efficacy, cost and effort parameters. The highest rank for each category is denoted by the number one (best), and the lowest denoted by the number eight (worst). Resulting quadrilaterals with larger areas represent better overall methods, while smaller quadrilaterals are less desirable

A ranked comparison of roller swab processing methods indicated that the roller trim method was the best approach, ranking lowest (most favourably) for efficacy and cost, and intermediate for effort (Fig. 2). The filtration method, although a relatively cost-effective approach yielding acceptable qPCR results but with low DNA concentration, required substantial time effort for sample processing. Buffer-based methods, while being considerably less labour-intensive, were costly and produced mediocre qPCR results and DNA concentrations.

Methods utilising the magnetic bead-based extraction protocols were ranked higher for qPCR results, DNA concentration, cost and effort, except for the DNA concentration for roller trim methods (Fig. 2).

## Discussion

All methods evaluated here were successfully applied to the collection and extraction of mammalian eDNA from small size surface rollers, however the roller trim method produced the best results and the magnetic bead-based Kingfisher extraction method consistently performed better than column-based Qiacube extractions. Considering financial, time and logistical constraints of a particular study together with the efficacy of eDNA detection allows for optimisation of eDNA protocols and is important for developing efficient and consistent methods for use in future studies.

Continuous improvement of techniques for next-generation biomonitoring is vital to ensure maximised accuracy [13], minimisation of cost and effort [14] and improved accessibility to the scientific and non-scientific community [15]. Differences in the quality of eDNA extracts between processing methods highlight the importance of optimising protocols for the desired substrate. In this study, methods which used aqueous solutions to wash eDNA from rollers prior to extraction (filtration and buffer methods) resulted in lower qPCR efficiency and DNA concentrations. This indicated that DNA may remain on rollers even after washing, and that there may be benefits to processing roller material directly, such as in the roller trim method.

Substantial variation between total DNA concentration and subsequent Ct values was present in this study, showing the varying effectiveness of each method in extracting both target and non-target DNA. For example, although the buffer method with the 20-min digest had higher total DNA concentrations than both the filtration and buffer with the 18-h digest, it had significantly higher Ct values, indicating that this processing method was less effective at isolating DNA from the target species. Longer digest periods may increase the amount of intracellular DNA available for amplification [16], and optimising of the digest time would aid in maximising the amount of target DNA for qPCR.

Higher total DNA concentrations and better qPCR efficiency from the magnetic bead-based Kingfisher protocol indicated that there may be processes within the column-based extraction protocol that result in lower quality and quantity of extracted DNA. Some DNA may be washed off and/or remain bound to the silica column, resulting in small amounts of total DNA loss [17]. Despite this, column-based extraction methods still provided adequate DNA extractions for downstream processing. The higher efficiency of magnetic bead extraction methods, particularly the Kingfisher’s high throughput extractions allows a larger number of samples to be processed simultaneously, reducing the effort required per sample. As such, magnetic bead approaches may be particularly appropriate for larger projects with hundreds of samples. For smaller studies with fewer samples, it may be more appropriate to use the Qiacube over the Kingfisher, which is designed to accommodate the maximum capacity of the instrument (96 samples) and may result in wastage of consumables if this capacity isn’t reached.

Selecting the most appropriate processing and extraction methods for field studies using roller swabs may not always be directly related to cost, effort and quality of DNA extracts. The practicality of each technique for a specific study is an essential consideration. For example, when sampling in remote locations, cold storage may not be available [18], leading to potential degradation of eDNA on the rollers which may influence species detection rates [16]. The buffer method, where the swab is immediately added to a storage and lysis buffer, may improve retention of surface DNA on roller swabs [8] and so be better suited to some field situations. Investigation of the efficacy of this and other preservation methods, such as ethanol misting [8] or desiccation of samples [19], is required for determining the optimal technique for preserving eDNA on roller swabs when immediate cold storage in the field is logistically challenging.

The size of the area to sample and consequently of the rollers used may also impact the practicality of each processing method. For example, the conventional water filtration method may be the optimum approach for larger rollers used to sample comparatively large surfaces, such as tree trunks, vegetation patches and fallen logs [2, 3, 20]. Using trim or buffer methods in this scenario would be impractical due to the large volume of buffer required for each method, which would substantially increase costs. For smaller surfaces, including tree hollows [4], nest boxes and other relatively small habitats, the use of trim or buffer methods are practical in terms of cost and logistics.

**Fig. 3:**
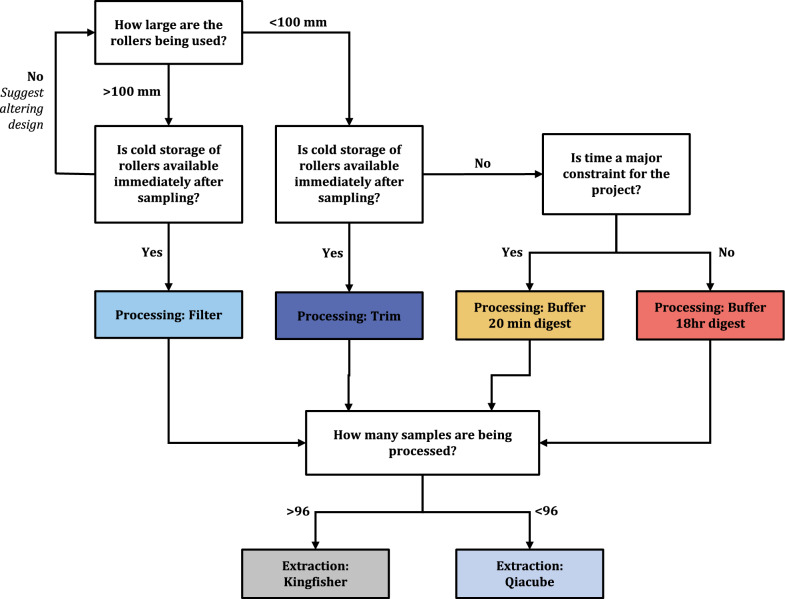
Decision tree for selecting the most practical processing and extraction methods for studies using roller swabs. Note that this does not consider data regarding quality and quantity of DNA produced, or the time, effort and cost parameters

From our findings we present here a decision tree to aid selection of the most appropriate processing and extraction method for extraction of eDNA from roller samples (Fig. 3).

## Limitations

This study was performed with a single assay on samples from a highly modified urban environment. There are many other different preservation and extraction buffers available and further investigation may increase the efficacy of buffer-based extraction methods.

### Supplementary Information

**Additional file 1: Figure S1.** Overview of roller processing and extraction methods. Note the buffer processing method was split into a 20 minute digest and and 18 hour digest by subsampling the initial buffer solution that rollers were submerged in. **Figure S2.** Approximate sample costs for processing and extraction methods for eDNA extracts from surface roller swabs. Costs calculated using current pricing as of late 2023 for reagents, consumables and equipment. Note costing does not include labour or initial capital costs of extraction equipment including Qiacube (Qiagen) or KingFisher Flex (ThermoFisher Scientific). All pricing in Australian Dollar (AUD). **Figure S3.** Approximate time for processing and lysis (digest) of surface roller swabs between four processing methods (filter, trim, buffer 20min and buffer 18hr) and two extraction methods. (Qiacube and Kingfisher). Note that the digest time has minimal impact on total effort, as it requires no additional involvement by the researcher, however it is important to consider this if sample turnaround is an important factor.

## Data Availability

The datasets analysed during this study are available from the corresponding author on reasonable request.

## Abbreviations

eDNA: Environmental DNA
qPCR: Quantitative polymerase chain reaction
Ct: Cycle threshold
