# Supplementary material for: On a roll: a direct comparison of extraction methods for the recovery of eDNA from roller swabbing of surfaces
Source: BMC Res Notes. 2023 Dec 18;16:370. doi: 10.1186/s13104-023-06669-5 (PMC10726604; doi:10.1186/s13104-023-06669-5)
Supplement: Supplementary file 1 — Additional file 1: Figure S1. Overview of roller processing and extraction methods. Note the buffer processing method was split into a 20 minute digest and and 18 hour digest by subsampling the initial buffer solution that rollers were submerged in. Figure S2. Approximate sample costs for processing and extraction methods for eDNA extracts from surface roller swabs. Costs calculated using current pricing as of late 2023 for reagents, consumables and equipment. Note costing does not include labour or initial capital costs of extraction equipment including Qiacube (Qiagen) or KingFisher Flex (ThermoFisher Scientific). All pricing in Australian Dollar (AUD). Figure S3. Approximate time for processing and lysis (digest) of surface roller swabs between four processing methods (filter, trim, buffer 20min and buffer 18hr) and two extraction methods. (Qiacube and Kingfisher). Note that the digest time has minimal impact on total effort, as it requires no additional involvement by the researcher, however it is important to consider this if sample turnaround is an important factor. [file 13104_2023_6669_MOESM1_ESM.docx]

Additional information for

**On a roll – a direct comparison of extraction methods for the recovery of eDNA from roller swabbing of surfaces.**

***Additional Methods***

Single-use 50 mm microfibre rollers were decontaminated by submersion in a 10% bleach solution (White King Premium Bleach, ∼5% sodium hypochlorite) for 10 minutes, thoroughly rinsed with deionised water and allowed to dry in a sterile fume hood. Rollers were further decontaminated in a UV cabinet for 15 minutes before being stored in zip lock bags until use. Roller frames were decontaminated prior to sampling by submersion in 100% bleach (White King Premium Bleach, ∼5% sodium hypochlorite) for 10 minutes before being thoroughly rinsed in deionised water.

A dog specific primer assay CYTBCA3-kh was used (Kurniasih et al., 2020), targeting a 105 base pair region of the cytochrome B gene with the primers CYTBCA-kh_F (5’-CCT TAG CCA ATG CCT ATT C-3’) and CYTBCA-kh_R (5’-GCG ACT TGT CCG ATA ATG-3’). *In silico* testing of the assay against common lab contaminants and potential field contaminants confirmed its specificity in detecting only domestic dog DNA.

DNA extracts were amplified on a StepOnePlus real-time PCR system (Applied Biosystems, Massachusetts, USA) under the following conditions: 95°C for 5 minutes and 50 cycles of 95 °C for 30 seconds, 58°C annealing temperature for 30 seconds and 72°C for 45 seconds. A melt curve of 95°C for 15 seconds, 60°C for one minute and 95°C for 15 seconds was used, ending with a ten-minute elongation at 72°C. Each 25 μL qPCR mix contained: 2.5mM MgCl2 (Applied Biosystems), 1× PCR Gold buffer (Applied Biosystems), 0.25 mM dNTPs (Astral Scientific, Australia), 0.4 mg/mL bovine serum albumin (Fisher Biotec, Australia), 0.4 μmol/L forward and reverse primer, 1 U AmpliTaq Gold DNA polymerase (Applied Biosystems) and 0.6 μL of a 1:10,000 solution of SYBR Green dye (Life Technologies, USA). Cycle-threshold (Ct) values were recorded for all samples and all PCR reactions were performed in duplicate and a single 1/10 dilution was included per sample. Positive controls using synthetic dog DNA were included at multiple dilutions (1 ng/µL, 0.1 ng/µL and 0.01 ng/µL), containing a 1000 bp region of the mitochondrial cytochrome B gene, and non-template positive controls (Quenda, Black Cockatoo and Tiger Snake) were added to confirm primer specificity. Extraction controls and non-template controls were included in qPCR runs.


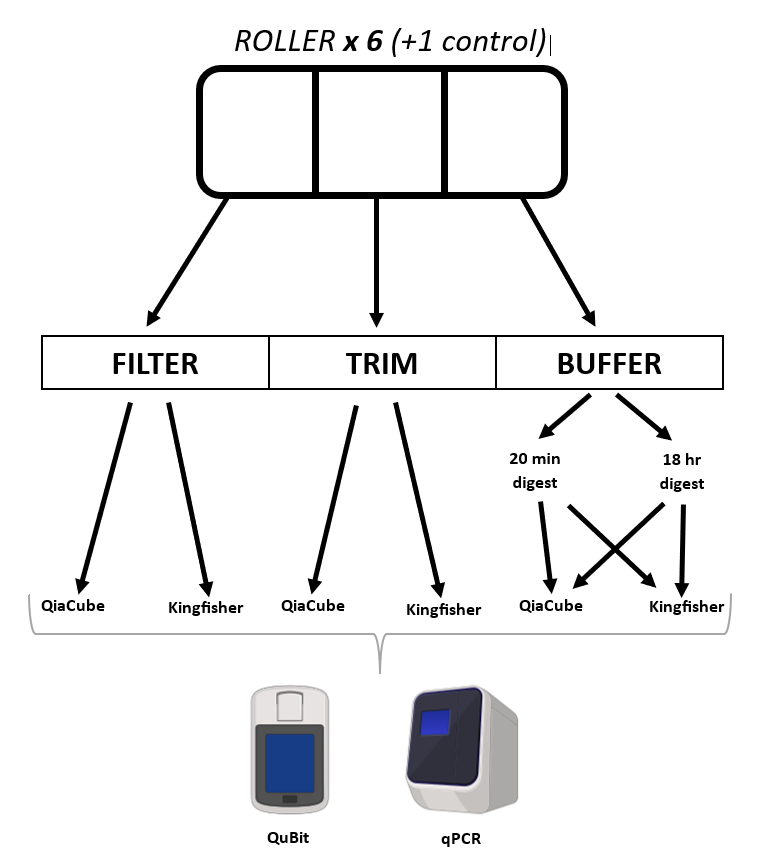


**Figure S1.**  Overview of roller processing and extraction methods. Note the buffer processing method was split into a 20 minute digest and and 18 hour digest by subsampling the initial buffer solution that rollers were submerged in.


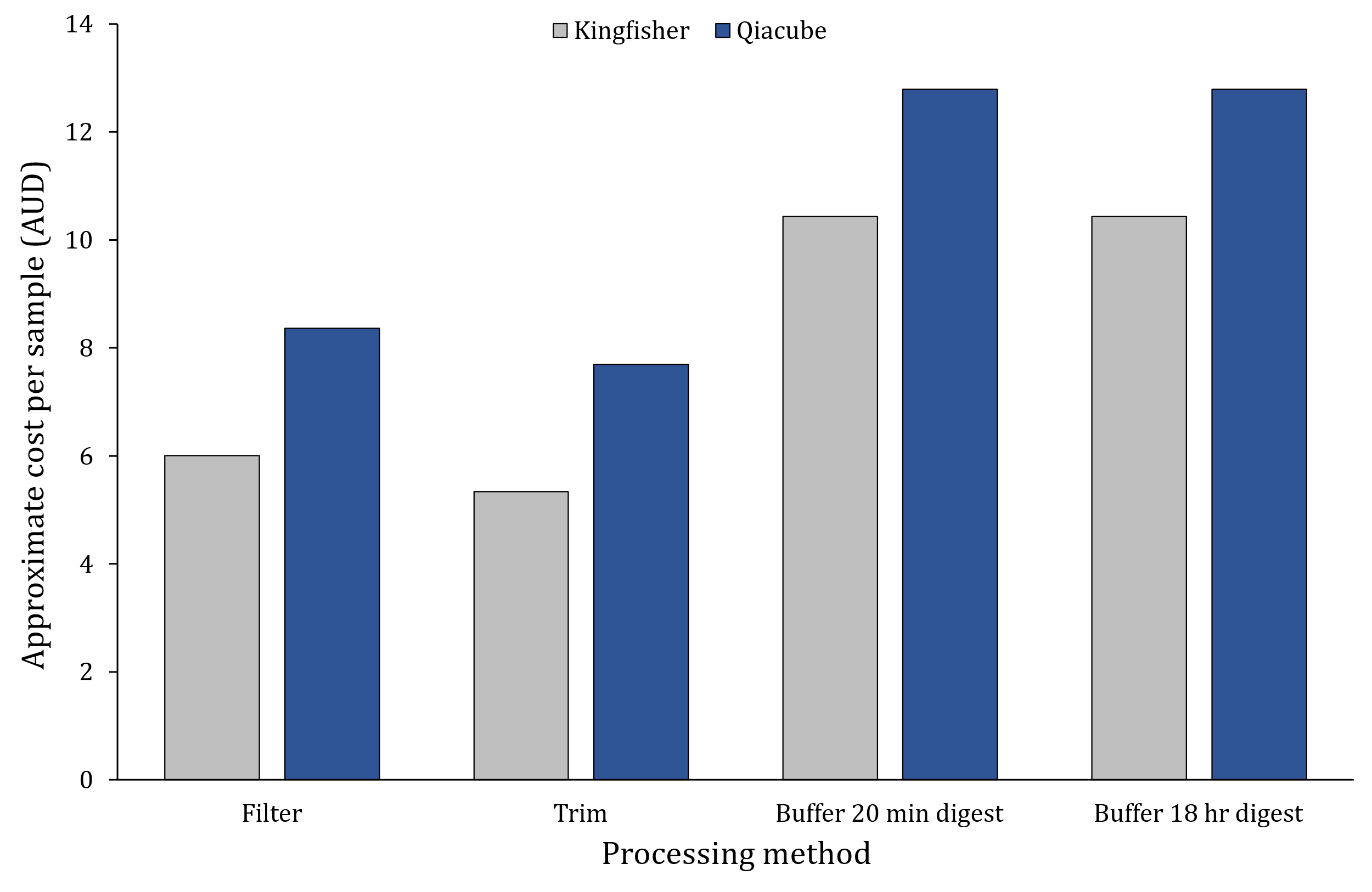


**Figure S2.**  Approximate sample costs for processing and extraction methods for eDNA extracts from surface roller swabs. Costs calculated using current pricing as of late 2023 for reagents, consumables and equipment. Note costing does not include labour or initial capital costs of extraction equipment including Qiacube (Qiagen) or KingFisher Flex (ThermoFisher Scientific). All pricing in Australian Dollar (AUD).


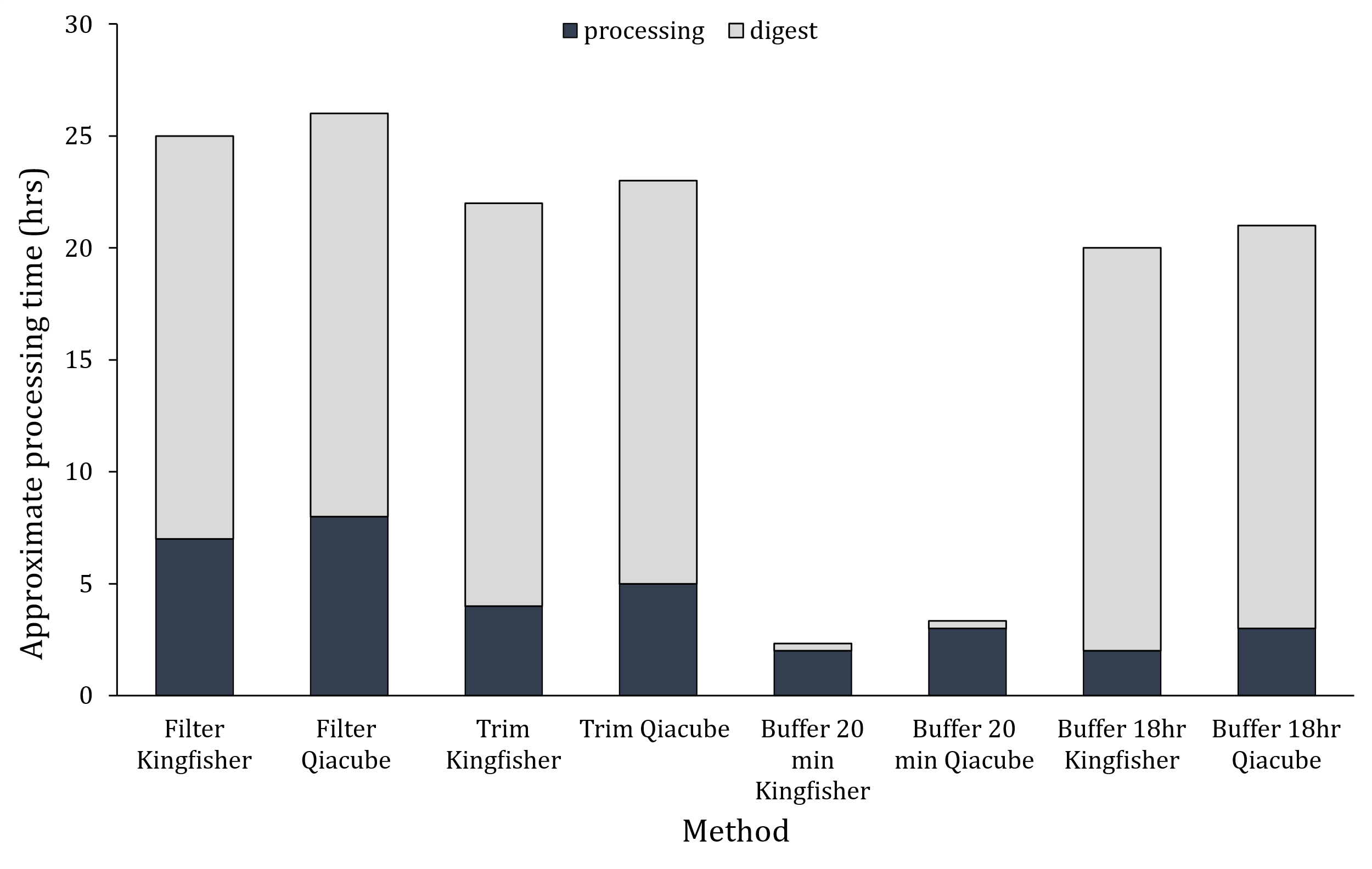


**Figure S3.** Approximate time for processing and lysis (digest) of surface roller swabs between four processing methods (filter, trim, buffer 20min and buffer 18hr) and two extraction methods. (Qiacube and Kingfisher). Note that the digest time has minimal impact on total effort, as it requires no additional involvement by the researcher, however it is important to consider this if sample turnaround is an important factor.
